# RecQ helicases in DNA repair and cancer targets

**DOI:** 10.1042/EBC20200012

**Published:** 2020-10-23

**Authors:** Joseph A. Newman, Opher Gileadi

**Affiliations:** Structural Genomics Consortium, University of Oxford, Oxford OX3 7DQ, U.K.

**Keywords:** DNA damage, DNA synthesis and repair, protein structure, synthetic lethality

## Abstract

Helicases are enzymes that use the energy derived from ATP hydrolysis to catalyze the unwinding of DNA or RNA. The RecQ family of helicases is conserved through evolution from prokaryotes to higher eukaryotes and plays important roles in various DNA repair pathways, contributing to the maintenance of genome integrity. Despite their roles as general tumor suppressors, there is now considerable interest in exploiting RecQ helicases as synthetic lethal targets for the development of new cancer therapeutics. In this review, we summarize the latest developments in the structural and mechanistic study of RecQ helicases and discuss their roles in various DNA repair pathways. Finally, we consider the potential to exploit RecQ helicases as therapeutic targets and review the recent progress towards the development of small molecules targeting RecQ helicases as cancer therapeutics.

## Introduction

**Figure 1:**
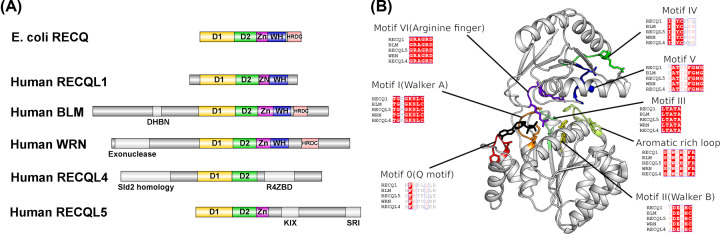
Sequence and domain organization of the human RecQ helicase family (**A**) Domain organization of select bacterial and human RecQ helicases. (**B**) Position and sequences of the conserved helicase motifs mapped onto the catalytic core structure.

The RecQ family of helicases unwind DNA in a 3′ to 5′ direction and contribute to the maintenance of genome integrity by playing important roles in multiple DNA repair pathways. Single-celled organisms and lower eukaryotes generally contain a single RecQ helicase, whilst human cells contain 5 RecQ family proteins: RECQL1, Bloom’s syndrome helicase (BLM), Werner syndrome helicase (WRN), RECQL4 and RECQL5. RecQ family members feature a conserved helicase core, comprising both N-terminal (D1) and C-terminal (D2) helicase lobes that share similarities with other superfamily 2 (SF2) helicases, and a RecQ specific C-terminal (RQC) domain that varies amongst the individual family members (Figure 1A). Mutations in the RecQ family genes BLM and WRN are linked to rare disorders associated with genome instability, premature ageing and cancer predisposition named Bloom’s syndrome [1] and Werner’s syndrome [2], respectively. Mutations in RECQL4 give rise to three related syndromes, Baller–Gerold syndrome, RAPADILINO and Rothmund–Thomson syndrome [3].

## RecQ helicases in DNA repair pathways

A gradually emerging picture for the human RecQ family is that each family member is involved in multiple pathways of DNA repair, mediated by a diverse array of protein interactions. Some roles appear to be shared by multiple family members, whilst others appear to be unique, as demonstrated by the cellular phenotypes of the various RecQ associated syndromes.

## Homologous recombination

BLM has been found to play multiple roles in the homologous recombination (HR) pathway. In the initial stages of the HR pathway, BLM is capable of interacting with EXO1 [4] and DNA2 [5,6] and promoting long range end resection, whilst in the latter stages it is capable, along with other RecQ family members of Holiday junction (HJ) branch migration [7,8], and in a complex with Topoisomerase Iiα and RMI1/2 plays a key role in the dissolution of double HJ [9]. This is a reaction that resolves double HJ without the exchange of genetic material between sister chromatids, and explains the increased frequency of sister chromatid exchanges found in BS cells [10]. BLM also plays a regulatory role in HR by disrupting RAD51 nucleoprotein filaments [11], an activity that is also shared by RECQL5 [12].

## Canonical nonhomologous end joining

Canonical nonhomologous end joining (c-NHEJ) is a template-independent repair process that relies on the DNA end recognition heterodimer Ku70/80, a DNA dependent protein kinase catalytic subunit (DNA-PKcs) and a Ligase4–XRCC4–XLF repair complex. WRN, which is unique in the RecQ family in having a 3′ to 5′ exonuclease domain in its N-terminus, has been found to interact with Ku via motifs on its N and C-terminus [13–15], with the interaction stimulating the WRN exonuclease activity [13]. WRN has also been found to interact with and become phosphorylated by DNA-PKcs, an interaction that stimulates WRN helicase but not nuclease activity [16,17]. This interaction may also be mediated via Ku [18], which is a common interacting partner. WRN phosphorylation by DNA-PKcs appears to inhibit helicase activity and also counteract the stimulation of the nuclease activity conferred by the Ku interaction [18], and influences the re-localization of WRN to the nucleolus after accumulation at damage sites [17]. Whilst WRN is not a core component of the c-NHEJ pathway it appears to be important for efficient double strand break repair [17], and is involved in pathway choice promoting classical NHEJ over alternate pathways via shielding DNA ends from resection [19].

## Telomere maintenance

The interplay between WRN and DNA-PKcs may play a role in telomere maintenance, where the interaction was found to selectively stimulate WRN helicase activity on model telomeric D-loop substrates [16]. Further evidence for involvement of WRN in telomere maintenance comes from the fact that WRN and BLM have been found to interact with members of the core shelterin complex POT1 [20] and TRF2 [21,22], and these interactions stimulate the helicase activity. The helicase activity of WRN has been shown to be required for telomere replication by lagging strand synthesis [23], and defects in this process may explain some of the genome instability and clinical features exhibited in WS [24]. WRN and BLM also appear to be involved in the alternative lengthening of telomeres (ALT) pathway, that is a telomerase independent pathway, that uses HR to lengthen telomeres and is frequently activated in cancer [25].

## Replication stress

Replication forks that stall upon meeting a barrier are a major cause of genome instability, and failure to stabilize, repair and restart can lead to fork collapse leading to genome rearrangements, cell death and disease. Both RECQL1 and RECQL4 have been found to associate with replication origins [26], with RECQL4 being particularly important for replication initiation where it promotes loading of other replication factors [27], although it is not clear what role the helicase activity of RECQL4 plays in this process [28]. RECQL1 appears to play a role in repair of stalled or collapsed replication forks, and is enriched at common fragile sites upon replication stress [29]. Stalled replication forks can be stabilized by fork reversal, a process whereby the nascent leading and lagging strands anneal to each other to create a chicken foot like structure containing a holiday junction. The formation of reversed forks is dependent on RAD51 and stalled forks are stabilized by PARP1 [30]. RECQL1 has been found to be a key factor in catalyzing replication fork restart and this activity is inhibited by PARP-1 [31]. RECQL5 has also been found to disrupt RAD51 filaments on stalled replication forks after reverse branch migration by RECQL1 [32], facilitating nuclease fork cleavage by nucleases, including MUS81-EME1 with which it physically interacts [33].

## Transcription replication conflicts

RECQL5 is unique amongst the human RecQ helicases in playing a direct role in transcription, achieved by its association with RNA polymerases I and II [34,35]. In the case of RNA polymerase II this interaction is formed by a discrete helical domain in the C-terminus of RECQL5 that interacts in a manner resembling TFIIS [36], and appears to contribute towards genome integrity by inhibiting transcript initiation and elongation and preventing transcription-replication collisions in actively transcribed regions [37,38]. Further roles for RECQL5 in transcription replication conflicts revolve around its ability to promote chromatin remodeling complexes and dislodge RNA polymerase II from DNA [39], or its role in disrupting RAD51 filaments inhibiting fork reversal and promoting restart [32]. BLM has also been found to play a role in the early stages of transcription replication conflicts, being rapidly recruited along with BRACA2 and FANCD2 to damage foci upon exposure to transcription stalling drugs, whilst depletion of BLM rendered cells hypersensitive to these agents [40]. This localization is dependent on the BLM association with FANCD2 [41], and the role of BLM in the early transcription replication conflict response appears to involve its helicase activity [40].

## Structures of RecQ helicases

**Figure 2:**
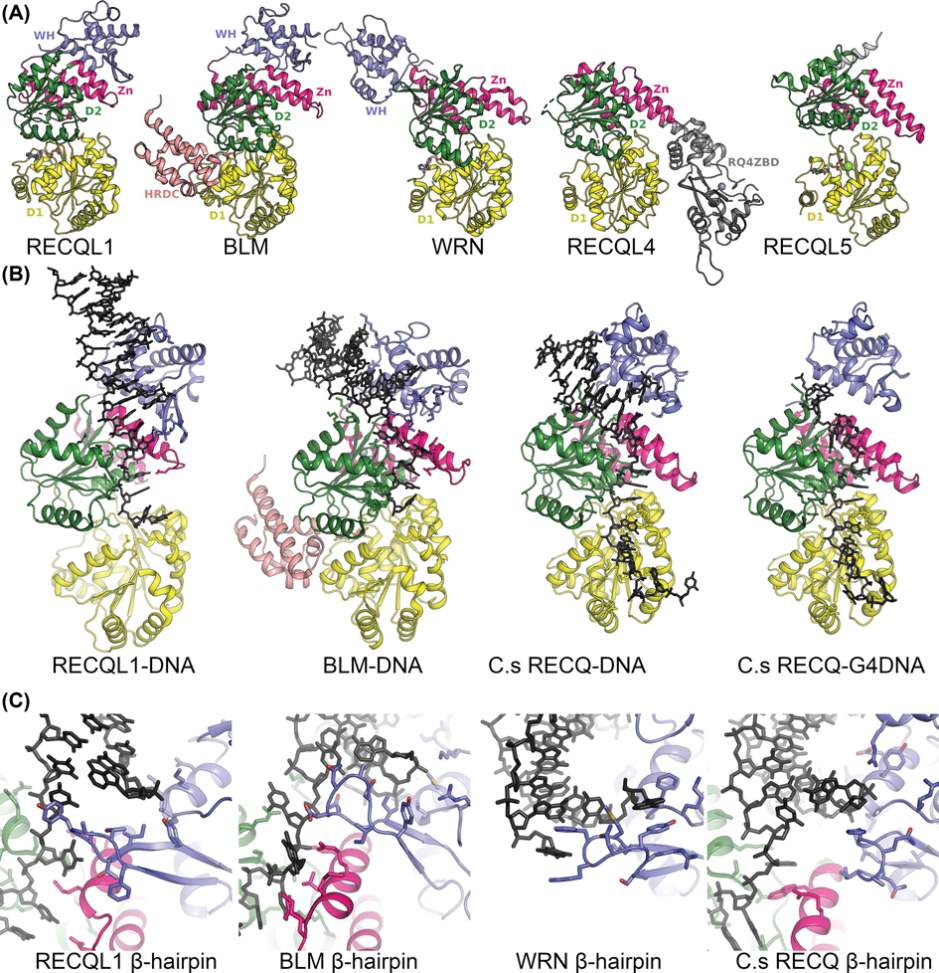
Structures of RecQ helicases (**A**) Structures of the five human RecQ family members RECQL1 [43], BLM [46], WRN [49], RECQL4 [48] and RECQL5 [47] viewed from the same orientation, with domains color coded as for Figure 1A. (**B**) Structures of current RecQ DNA complexes RECQL1-DNA [44], BLM-DNA [46], C.s RECQ-DNA [57] and C.s RECQ-G4DNA [58]. (**C**) Close up view of the interface between the WH β-hairpin and DNA. In RECQL1 [44] and WRN [52], aromatic residues on the β-hairpin make stacking interactions with unpaired DNA bases, whilst the β-hairpins on BLM [46] and *C. sakazakii* RECQ [57] are shorter and more polar.

In the last 15 years, the structural understanding of the human RecQ helicase family has increased massively such that structures are now available for the helicase core of the entire family (Figure 2A). Early structural work focused on bacterial RecQ proteins [42], whilst the first human RecQ catalytic core structure to be determined was the RECQL1 catalytic core in 2009 [43]. This was followed several years later by RECQL1 DNA complex structures [44]. BLM helicase structures were determined firstly in complex with DNA in 2014 [45] and in 2015 in complex with DNA and also a nanobody complex [45,46]. In 2017, the RECQL5 helicase core structures were determined in two different conformations in the presence and absence of nucleotide [47]. In the same year, the structure of RECQL4 was determined containing the helicase core and a novel RECQL4 specific C-terminal domain [48]. Finally, the structure of WRN helicase domain was deposited to the PDB in late 2019 to complete the structural coverage of the entire human RecQ family [49]. These studies have been complemented by parallel structural efforts on isolated domains from RecQ helicases that either are helicase associated [50–52], or perform other specialized functions [53].

The common catalytic helicase core contains two domains (D1 and D2) that feature a common fold first identified in the *Escherichia coli* RecA protein [54] and are conserved amongst a wide selection of proteins including helicases, translocases, AAA+ motor proteins and the ABC transporter family. A pioneering bioinformatics analysis identified a series seven of conserved helicase motifs (I–VII) within these domains which was used to classify helicases into their six super-families and still to this day serves as a means to understand helicase structure and function [55,56]. Both domains feature a mixed seven stranded β-sheet flanked on either side by helices, with the nucleotide binding site being formed by clusters of conserved residues at the interface of the two domains (Figure 1B). Following closely from the D2 domain in most RecQ family members is the RecQ C-terminal (RQC) domain that is a combination of a four cysteine Zn^2+^ binding subdomain, helical hairpin and a DNA-binding winged helix (WH) domain. The WH domain appears to be loosely associated with the rest of the helicase core, adopting variable conformations in the absence of DNA, whilst being more consistent in the various DNA complexes where it makes extensive interactions with the junction between double and single-stranded regions [44–46,57]. Overall, the RQC domain is less well conserved across RecQ family members and features variability in the length of the helical hairpin, the topology of the Zn^2+^ binding region, and the extent and character of the β-hairpin. In addition, for human RECQL4 and RECQL5 only part of the RQC region is present, with RECQL5 containing the helical hairpin and Zn^2+^ subdomains but not a WH domain, instead a single α-helix occupies a similar position and appears to be essential for helicase activity [47]. The RQC domain of RECQL4 is significantly different to any other human RecQ helicase containing a unique insertion of a RECQL4 Zn^2+^-binding domain, which is inserted in between the N and C-terminal lobes of the helical hairpin, and features a zinc-binding domain (three cysteine and one histidine) and two domains that feature similarities to WH domains, which are distinct from WH domains in other RecQ helicases [48] (Figure 2B).

## DNA complex structures

Structures of RecQ DNA complexes have been determined so far for RECQ1 [44], BLM [45,46] and Bacterial RecQ enzymes [57,58] as well as the isolated WRN WH domain [52]. These structures have been obtained with similar DNA substrates (double-stranded DNA with single-stranded 3′ overhangs) and the interfaces show several conserved features. The WH domain makes the majority of contacts to the double-stranded DNA, whilst the single-stranded region forms contacts to conserved helicase motifs IVa and V on the 2nd RecA domain (Figure 2B). Another conserved feature of the DNA protein interface is an extended β-hairpin (the wing of the WH domain) which is positioned at the interface between the double- and single-stranded DNA and makes contacts to unpaired bases in the junction. Mutational analysis of RECQL1 demonstrated that a single aromatic residue Y564 which forms at the tip of the β-hairpin is essential for helicase activity, and functions as a strand separation pin [43]. Similar aromatic stacking interactions were found in a WRN WH DNA complex with two aromatic residues Y1034 and F1037 forming interactions with unpaired bases on both sides of the junction [52]. In contrast both BLM and bacterial RecQ helicases feature a significantly shorter β-hairpin (Figure 2C) that makes polar interactions with the DNA junction, and in the case of the *E. coli* enzyme at least, the β-hairpin is not required for helicase activity [43]. In the absence of such contacts, it has been suggested that RecQ helicases might facilitate strand separation by binding DNA with an increased break angle between double- and single-stranded regions, as has been observed when comparing bacterial RecQ and RECQL1 structures [57]. Such a mechanism may also be relevant to RecQ family members such as RECQL5 and RECQL4 that lack a canonical WH domain. Whilst the details of the DNA interfaces are not known for these enzymes, a mutational study on RECQL5 showed a single “wedge” helix, which occupies a similar position as the WH strand separation motifs, greatly enhances DNA binding and is required for helicase but not ATPase activity [47], suggesting it may play a similar functional role. The question of how RecQ helicases bind to more complicated DNA substrates such as holiday junctions, collapsed forks of G-quadruplexes remains largely unanswered, although a recent structure of a bacterial RecQ in complex with an unwound G-quadruplex suggested an unwinding mechanism that uses a base flipping mechanism and guanine specific pocket [58].

## RecQ helicase mechanism

**Figure 3:**
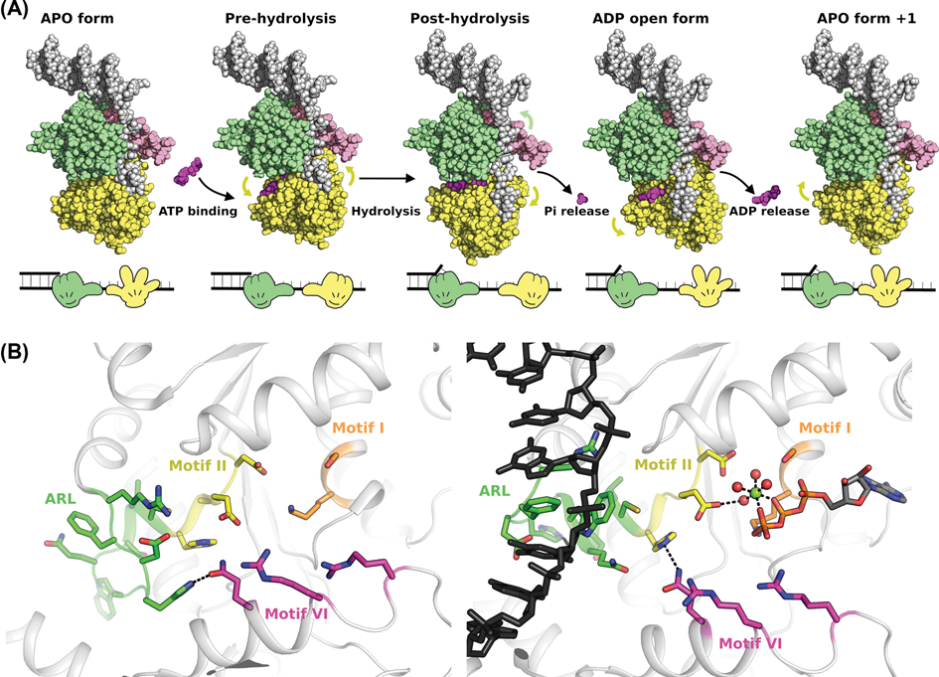
Model for the unwinding mechanism of RecQ helicases (**A**) Proposed inchworm style mechanism for RecQ helicase activity. The binding, hydrolysis and release of nucleotide induce conformational changes in the positioning of the two domains and can influence the affinity of D1 for DNA. The high and low affinity forms of D1 are depicted on schematic diagram on the lower panel as closed and open hands, respectively. (**B**) Close up view of the remodeling of the conserved helicase motifs in the active site following conformational changes (motifs are labeled and colored as for Figure 1B). The left-hand panel shows the active site in the absence of nucleotide, with polar contacts formed between conserved residues in motif VI and the ARL, presumably stabilizing the extended coil conformation. Binding of ATP induces a helix-to-coil transition in motif I that re-orientates the two domains, allowing the ARL to transition to a more helical conformation and interact strongly with DNA. This interaction causes a small shift in motif II allowing a more optimal positioning of the catalytic glutamate on motif II to activate a water molecule for nucleophilic attack.

Both single molecule and kinetic analysis of various RecQ helicases indicate that they may share a conserved reaction mechanism with one base unwound per ATP consumed, together with moderate reaction rates (50–100 nucleotides unwound per second) and processivity (50 nucleotides per encounter) [59–61]. Structural studies have generally provided support for an “inchworm” type mechanism of DNA translocation where one of the two RecA helicase lobes remains attached to the DNA at all times and the enzyme cycles between high and low affinity states, accompanied by relative movements of the two domains that provide directional tracking along one of the DNA strands (Figure 3A). A requirement for this mechanism is that binding, hydrolysis and release of nucleotide induce conformational changes in the positioning of the two domains and also induces switching from high affinity to low affinity DNA-binding states. Systematic comparisons of the relative positioning of the two RecA of the various RECQ family structures reveal significant variations in inter domain positioning that appear to be linked to the nucleotide bound status of the complex [47], and could represent different states of the catalytic cycle. A further key insight into the mechanism of RecQ helicases was revealed by the structure of a DNA complex of RecQ from the gram-negative bacterium *Cronobacter sakazakii*, which revealed detailed DNA interactions formed by a conserved aromatic rich loop (ARL) found in the D1 domain of RecQ and related SFII helicases [57,62]. Importantly, this loop adopts a different conformation in the absence of DNA, and the close proximity of the ARL to the Walker B motif (helicase motif II) suggests a mechanism by which the binding to DNA creates a rearrangement of the active site, positioning the catalytic glutamate in a favorable position to perform ATP hydrolysis (Figure 3B) [57].

These observations together with mutagenesis studies and comparisons of APO and nucleotide bound RecQ structures have allowed the proposal of a molecular mechanism for the family [47]. In this model, in the absence of nucleotide the ARL makes polar contacts to conserved helicase motifs on D1, preventing it adopting the alternate conformation and ensuring a compact conformation of the two domains. Binding of ATP disrupts these polar contacts allowing the domains to move apart, and the ARL to become remodeled to bind tightly to DNA. This in turn causes a small shift in the catalytic walker B motif that becomes optimally positioned to stimulate ATP hydrolysis (Figure 3B). Subsequent steps in the mechanism are more speculative, but it is assumed that the “power stroke” may allow D2 to advance a single step along the DNA tract, possibly due to interactions mediated via the γ-phosphate sensing Arginine fingers (part of helicase motif VI), and that phosphate release precedes ADP release and may trigger destabilization of the ARL DNA contacts. An open ADP bound conformation that was observed for RECQL5 and *Deinococcus radiodurans* RecQ [47,63] may be required for nucleotide release enabling the compact APO form to form once again.

## Function of the HRDC domain

**Figure 4:**
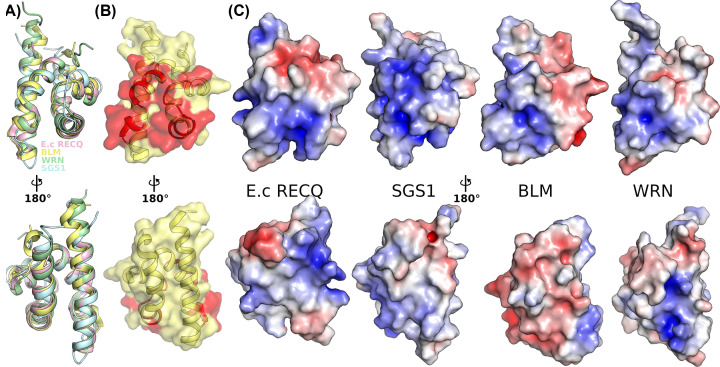
Structure of the HRDC domain (**A**) Superposition of the HRDC domains from *E. coli* RECQ [51], *S. cerevisiae* SGS1 [50], and human BLM [46] and WRN [65]. (**B**) Surface view of the BLM HRDC domain [46] with the regions making contacts with the BLM RecA core highlighted in red. The top panel shows a view of the contact surface whilst the bottom panel shows the opposite face (same views shown throughout). (**C**) Surface electrostatics representation of the HRDC domains from *E. coli* RECQ [51], *S. cerevisiae* SGS1 [50], and human BLM [46] and WRN [65]. The plots are contoured at ± 5 *KT/e* with positive regions in blue and negative red. The positive charge on the RecA contacting surface is conserved whilst the positively charged external surface is not conserved in human BLM.

The Helicase and RNAse D C-terminal (HRDC) domain is present in only a subset of the RecQ family that includes BLM and WRN in the human enzymes together with lower eukaryotic and bacterial RecQ family members. The domain is a small 5 helical bundle that folds independently and is found C-terminal to the WH domain (Figures 1A and 4A) and does not appear to be required for the basic helicase activity on simple substrates. It was initially thought to comprise an accessory DNA-binding domain based on its conservation to other families of DNA-binding proteins and the identification of a positively charged surface on structures of the isolated domain from *E. coli*, which has DNA binding properties in isolation [51,64] (Figure 4C). Studies on HRDC domains from BLM and WRN did not find a charged surface and failed to demonstrate convincing DNA-binding activity for the domain in isolation [65,66], although in the case of BLM the HRDC domain was found to be essential for the double Holliday junction activity of the enzyme [67]. Structural studies of BLM helicase showed that the HRDC domain interacts with the helicase core (Figures 2A and 4B), making contacts with both D1 and D2 in the nucleotide bound form of the enzyme [45,46]. Importantly, these contacts appear to be dependent on the nucleotide bound state of the enzyme [46], suggesting that HRDC interactions with the RecA core may be part of the catalytic cycle of these enzymes. Kinetic studies on *E. coli* RecQ show that the HRDC domain suppresses single-stranded DNA dissociation, and fork unwinding regardless of its DNA-binding ability [68], and was found to stabilize DNA sequence dependent paused states of the enzyme [69], suggestive of an intrinsic recombination quality control mechanism. Further clues as to the diversification of functions of this domain comes from the fact that some bacterial RecQ proteins such as from *D. radiodurans* contain three HRDC domains, with the N-terminal HRDC domain critical for high affinity DNA binding, whilst the C-terminal domains attenuate the DNA-dependent ATP hydrolysis rate of the enzyme in an apparent separation of functions [70].

## Oligomeric status of RecQ helicases

Whilst the helicase domains of RecQ helicases tend to be monomeric, there are various reports of higher order oligomeric states of RecQ helicases such as tetrameric or hexameric WRN, BLM and RECQL1 species observed in electron microscopy studies [71–73]. These higher order structures are not required for helicase activities, nor do RecQ enzymes show cooperativity in ATPase activity. For RECQL1 and WRN, the higher order oligomer was observed to exist in conjunction with a lower order dimeric form that in RECQL1 appears to be mediated by associations of the WH domain [44,74]. The higher order RECQL1 oligomer is mediated by residues in the N-terminus and is associated with strand annealing activities of the enzyme [72], whilst the WRN oligomer was only observed in the presence of DNA [73]. BLM was also observed to exist in multiple oligomeric forms with the higher order form being observed to dissociate in the presence of ATP [71,75]. A conserved helical bundle in the N-terminus of BLM was shown responsible for dimer formation, forming a v-shaped helical bundle with antiparallel association of chains forming a packed hydrophobic core [75]. This dimeric module is then assumed to associate into tetramers or hexamers mediated by interactions at the extreme N-terminus of BLM. For RECQL5 SAXS studies in solution studies indicate the protein is most likely monomeric [36,47], although the proteins used in these studies were missing some residues in the C-terminus. For RECQL4 comparatively little is known about the oligomeric status of the full-length enzyme with the crystallized helicase domain being monomeric [48].

## Inhibition of RecQ helicases as cancer targets, rationale, current progress and challenges

The synthetic lethal approach to cancer therapy relies on exploiting complementary pathways that result in specific cell death in cancer cells due to the existing mutational background, and thus can afford a wide therapeutic window. The success of this approach in DNA repair pathways is evidenced by the current clinical successes with PARP inhibition. Because of their prominent roles in DNA repair pathways there is considerable interest in the development of new cancer therapeutics targeting RecQ helicases. Recently a striking synthetic lethal relationship has been identified in three independent studies between WRN helicase and microsatellite instability-high cancers [76–78]. WRN was the top dependency identified in a genome wide inactivation study and the helicase activity of WRN was demonstrated to be essential for survival of these cells but not related microsatellite stable cancer cells. Another recent study demonstrated an essential role for RECQL5 in triple-negative breast cancer (TNBC), a cancer that currently lacks an effective targeted treatment [79]. TNBC cells display high levels of endogenous DNA damage including replication stress and the generation of double strand breaks, and depletion of RECQL5 causes cell arrest *in vitro* and slows the growth of xenograft tumors *in vivo* [79]. Other less well-characterized relationships exist for the other RecQ family members with RECQL1, RECQL4 and BLM being significantly overexpressed in various cancers [80–82], with high expression generally being linked to poor prognosis, and depletion causing a reduction in proliferation and chemosensitization.

Whilst there are no currently approved or ongoing clinical trials for drugs specifically targeting RecQ helicases, there has been considerable effort to identify chemical classes and candidate small molecule inhibitors from academic labs. A high-throughput screen using a radiometric strand displacement assay was used to identify NSC19630 IC_50_ = 20 μM [83], which were also found to induce apoptosis in cells in a WRN-dependent manner [83,84]. Subsequent work identified a structural analogue NSC617145 [85] that induced sensitization to mitomycin C in cells carrying mutations in the Fanconi Anemia pathway. Whilst these compounds showed specificity over related helicases, concerns about the maleimide functional groups potential non-specific or covalent mode of action and the discovery of off target effects on unrelated enzyme classes [86] may explain the lack of development of these molecules towards clinical candidates. A fluorescence quenching DNA unwinding assay has allowed the screening of larger libraries and identification of further compound classes targeting WRN [87], these compounds display both reversible and irreversible modes of inhibition, with IC_50_ values in the low μM range although they only display limited selectivity over other helicases. Similar high-throughput screens have been used to identify BLM and RECQL1 inhibitors, although only compounds targeting BLM have been described in the literature [88,89]. The BLM inhibitor ML216 was the result of several iterations of chemical optimization and displays low μM activity towards BLM, and has activities on cells that induce sister chromatid exchanges, similar to the phenotype of Blooms syndrome cells [88]. The compound displays some selectivity over related helicases, although a significant low μM activity remains for WRN, limiting the utility of this compound as a chemical probe. A recent study found that isaindigotone derivatives, a naturally occurring alkaloid used in traditional Chinese medicine, are able to inhibit BLM with some improved properties compared to ML216 [90]. The isaindigotone compound was found to disrupt BLM binding to DNA, and displayed high affinity binding to BLM in ITC measurements (1.8 μM), as well as chemosensitization and antiproliferative effects on cells in a BLM-dependent manner [90], although the selectivity for this compound over related helicases was not reported.

Despite these efforts the RecQ family like the wider helicase superfamily family remain largely undrugged. One of the challenges associated with these targets is the high proportion of false positive hits obtained in typical high-throughput screening efforts, these can include aggregators, covalent modifiers or compounds that bind directly to DNA. Whilst these effects can be minimized by effective choice of assay parameters or counter-screening, hits from high-throughput screening should be viewed with some skepticism in the absence of further validation. One potential obstacle is that the helicase ATP-binding clefts appear to be less druggable than their kinase counterparts, whilst maintaining high levels of sequence and structural conservation that make selectivity a great challenge in this target class. Other modes of inhibition such as competition for DNA-binding sites may be challenging due to the highly polar nature of these interfaces. The lack of structural information on the binding modes RecQ targeting compounds has also hindered the development of current compounds, although this may change given the recent advancements in structural coverage of the family. It may be that innovative recent developments in drug discovery approaches such as fragment-based screening, DNA encoded libraries or targeted degradation (PROTAC) may be required to unlock this promising target class.

## Summary

- RecQ helicases have varying roles in a large number of DNA repair and genome maintenance pathways.
- Structures of human RecQ helicases solved over the last 15 years reveal a common helicase mechanism that involves conformational changes of the two helicase lobes driven by nucleotide binding hydrolysis and release.
- The HRDC domain is an accessory domain found in a subset of RecQ family proteins and appears to modulate core helicase activities amongst possible additional more specialized roles.
- RecQ helicases exhibit some catalytic activity as monomers but are found in a wide variety of oligomeric states, which may be required for their specific biological activities.
- RecQ helicases are an attractive target for the design of cancer therapeutics. Several academic labs have reported promising starting points for RecQ based drugs although the family remains undrugged.

## Open Access

Open access for this article was enabled by the participation of University of Oxford in an all-inclusive *Read & Publish* pilot with Portland Press and the Biochemical Society under a transformative agreement with JISC.

## Abbreviations

BLM: Bloom’s syndrome helicase
HRDC: Helicase and RNAse D C-terminal
RQC: RecQ specific C-terminal
TNBC: triple-negative breast cancer
WRN: Werner syndrome helicase
